# Rho-actin signaling to the MRTF coactivators dominates the immediate transcriptional response to serum in fibroblasts

**DOI:** 10.1101/gad.239327.114

**Published:** 2014-05-01

**Authors:** Cyril Esnault, Aengus Stewart, Francesco Gualdrini, Phil East, Stuart Horswell, Nik Matthews, Richard Treisman

**Affiliations:** 1 Transcription Group,; 2 Bioinformatics and Biostatistics Group,; 3 Advanced Sequencing Facility, Cancer Research UK London Research Institute, London WC2A 3LY, United Kingdom

**Keywords:** SRF, MRTF, TCF, Rho, signal transduction, chromatin

## Abstract

The transcription factor SRF (serum response factor) recruits two families of coactivators, the MRTFs (myocardin-related transcription factors) and the TCFs (ternary complex factors), to couple gene transcription to growth factor signaling. Here we investigated the role of the SRF network in the immediate transcriptional response of fibroblasts to serum stimulation. SRF recruited its cofactors in a gene-specific manner, and virtually all MRTF binding was directed by SRF. Much of SRF DNA binding was serum-inducible, reflecting a requirement for MRTF–SRF complex formation in nucleosome displacement. We identified 960 serum-responsive SRF target genes, which were mostly MRTF-controlled, as assessed by MRTF chromatin immunoprecipitation (ChIP) combined with deep sequencing (ChIP-seq) and/or sensitivity to MRTF-linked signals. MRTF activation facilitates RNA polymerase II (Pol II) recruitment or promoter escape according to gene context. MRTF targets encode regulators of the cytoskeleton, transcription, and cell growth, underpinning the role of SRF in cytoskeletal dynamics and mechanosensing. Finally, we show that specific activation of either MRTFs or TCFs can reset the circadian clock.

## Abstract (precis)

The transcription factor SRF (serum response factor) recruits two families of coactivators, the MRTFs (myocardin-related transcription factors) and the TCFs (ternary complex factors), to couple gene transcription to growth factor signaling. Esnault et al. investigate the role of the SRF network in the fibroblast serum response and demonstrate a critical role for MRTF signaling. MRTF genomic targets encode regulators of the cytoskeleton, transcription, and cell growth, underpinning the role of SRF in the cellular response to mechanical cues.

The SRF (serum response factor) transcription factor is an important regulator of cytoskeletal and muscle-specific gene expression (for reviews, see Posern and Treisman 2006; Olson and Nordheim 2010). SRF was first identified in studies of the fibroblast serum response, a classical model for cell cycle re-entry and wound healing. It functions in partnership with members of two families of signal-regulated cofactors: the MRTFs (myocardin-related transcription factors; MRTF-A, MRTF-B, and myocardin itself) and the TCF (ternary complex factor) family of Ets domain proteins (SAP-1, Elk-1, and Net). The MRTFs, which bind G-actin, respond to fluctuations in G-actin concentration induced by Rho GTPase signaling (Miralles et al. 2003; Vartiainen et al. 2007), while TCF activity is controlled by Ras–ERK signaling. However, the extent to which SRF is responsible for the serum-induced immediate transcriptional response and the roles played by its cofactors have remained uncharacterized.

The myocardin and TCF family proteins compete for a common surface on the SRF DNA-binding domain but also contact DNA flanking the SRF-binding site (Miralles et al. 2003; Wang et al. 2004; Zaromytidou et al. 2006). Classic genomic footprinting studies suggest that SRF constitutively binds DNA and thereby defines the targets for its cofactors (Herrera et al. 1989). Functional studies suggest that TCF and MRTF binding is gene-specific (Gineitis and Treisman 2001; Miralles et al. 2003; Wang et al. 2004), but the generality of this and its molecular basis remain to be determined. Where they have been compared, the similarity of MRTF and SRF inactivation phenotypes suggests that the MRTFs act solely through SRF (Medjkane et al. 2009; Mokalled et al. 2010). In contrast, the TCFs can act redundantly with other Ets proteins independently of SRF (for review, see Hollenhorst et al. 2011). Genomic studies revealed an association between SRF-binding sites and Ets, Sp1, AP-1, CREB, and NFY motifs (Valouev et al. 2008; Sullivan et al. 2011) and with CBP at signal-regulated enhancers (Kim et al. 2010).

Here we revisit the role of the SRF network in the fibroblast serum response and demonstrate a critical role for MRTF signaling. We identified >3100 SRF-binding sites, demonstrated that recruitment of SRF cofactors is indeed gene-specific, and showed that the majority of >2600 MRTF-binding sites exhibit MRTF-dependent SRF binding. We identified 960 serum-responsive SRF-linked genes and showed that most are controlled through MRTF signaling, which activates transcription by promoting both RNA polymerase recruitment and promoter escape. We defined candidate gene sets controlled by the MRTF and TCF families. Our data suggest that MRTF–SRF signaling is central to the serum response and strongly support the emerging idea that SRF signaling underlies the response of cells to mechanical cues.

## Results

### Much of SRF binding is controlled by Rho-actin signaling

**Figure 1.**
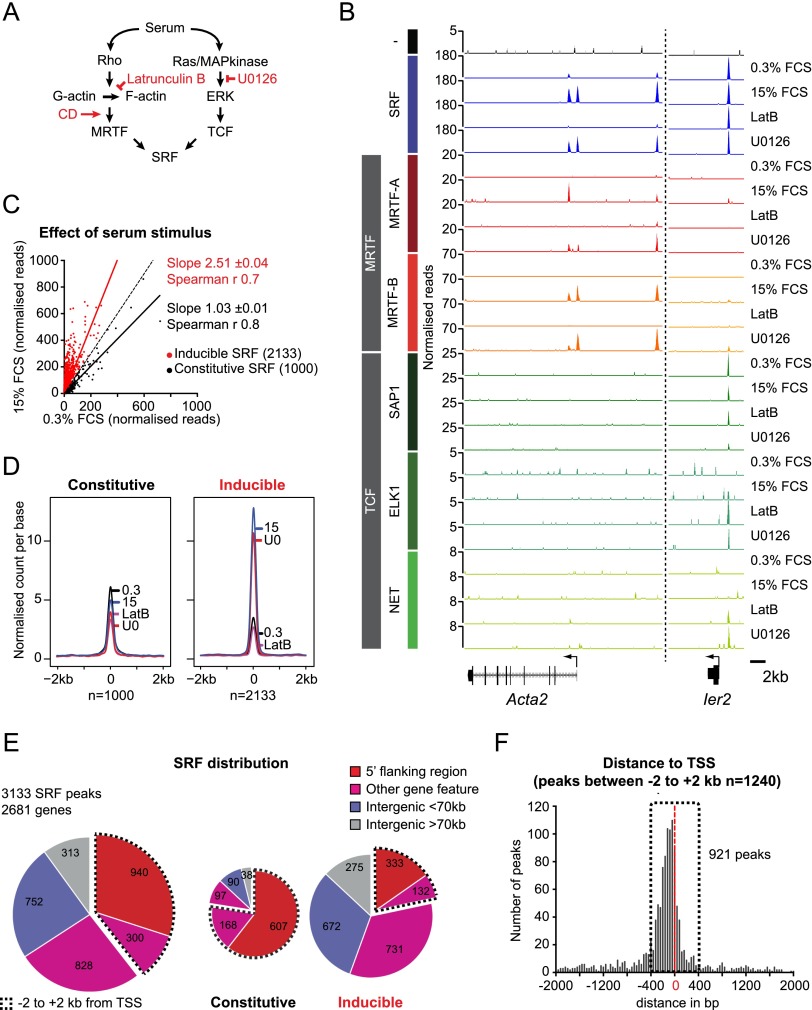
Much of SRF binding is controlled by growth factor signaling. (*A*) Signaling pathways, activators, and inhibitors affecting the SRF network. (*B*) Representative SRF, MRTF, and TCF cofactor-binding profiles as normalized reads per base pair. Cell culture conditions were resting cells (0.3% FCS), cells stimulated for 30 min (15% FCS), stimulated in presence of LatB (LatB), and stimulated in presence of U0126 (U0126). (*C*) Serum stimulation induces SRF binding. Scatter plot comparing ChIP-seq read counts in stimulated and resting cells. (Dotted line) Linear regression plot for all sites (Spearman *r*, 0.3; fold-inducibility, 1.7 ± 0.03). Note that division into inducible (>1.5-fold increase; red) and constitutive (<1.5-fold increase; black) populations greatly improves rank order correlations, respectively. Solid lines show linear regression plots for the two populations. (*D*) Metaprofile of SRF binding at constitutive and inducible sites. (*E*) SRF ChIP-seq peaks are associated with protein-coding genes (within ±2 kb of the TSS, *P* < 10^−999^; within a gene feature, *P* = 1.9 × 10^−106^; basic χ^2^ test). (Red) 5′ flanking sequences (−2 kb to the TSS); (pink) other gene features (5′ untranslated region [UTR], 338; introns, 740; coding exons, 39; 3′ UTR, 11); (blue) intergenic, <70 kb from TSS or pA site; (gray) intergenic, >70 kb from TSS or pA site. (*F*) Distribution of SRF sites around the TSS, shown as sites per 40-base-pair (bp) bin.

SRF represents the convergence point for serum-induced Rho-actin and Ras–ERK signals (Fig. 1A). We used chromatin immunoprecipitation (ChIP) combined with deep sequencing (ChIP-seq) to define genomic binding sites in fibroblasts for SRF, the MRTFs, and the TCFs and correlated binding with changes in gene transcription 30 min after serum stimulation, when SRF activation is maximal. To assess the role of SRF-linked signal pathways in regulation, we used the pathway-specific inhibitors Latrunculin B (LatB) and U0126, which inhibit Rho-actin and ERK signaling, respectively, and the MRTF-specific activator cytochalasin D (CD) (Supplemental Fig. S1).

We established a core set of 3133 SRF-binding sites by ChIP-seq (see the Materials and Methods). In contrast to expectations from classical studies of the c-fos gene (Herrera et al. 1989), the majority of SRF sites exhibited an increased ChIP-seq signal upon serum stimulation (Fig. 1B). Using an inducibility threshold of 1.5, we divided the SRF sites into 1000 “constitutive” and 2133 “inducible” sites, with mean inducibility 1.03 ± 0.01 and 2.51 ± 0.04, respectively (Fig. 1C; see the Materials and Methods). LatB, but not U0126, specifically inhibited SRF recruitment to inducible sites, suggesting that inducible SRF binding reflects MRTF recruitment (Fig. 1D; Supplemental Fig. S2A,B; see below). Almost 70% of SRF sites were associated with the immediate 5′ flanking region (−2 kb to the transcription start site [TSS]) or other gene features, and constitutive sites were selectively enriched within 2 kb of the TSS (Fig. 1E,F; Supplemental Table S1).

### MRTF binding is predominantly directed by SRF in fibroblasts

**Figure 2.**
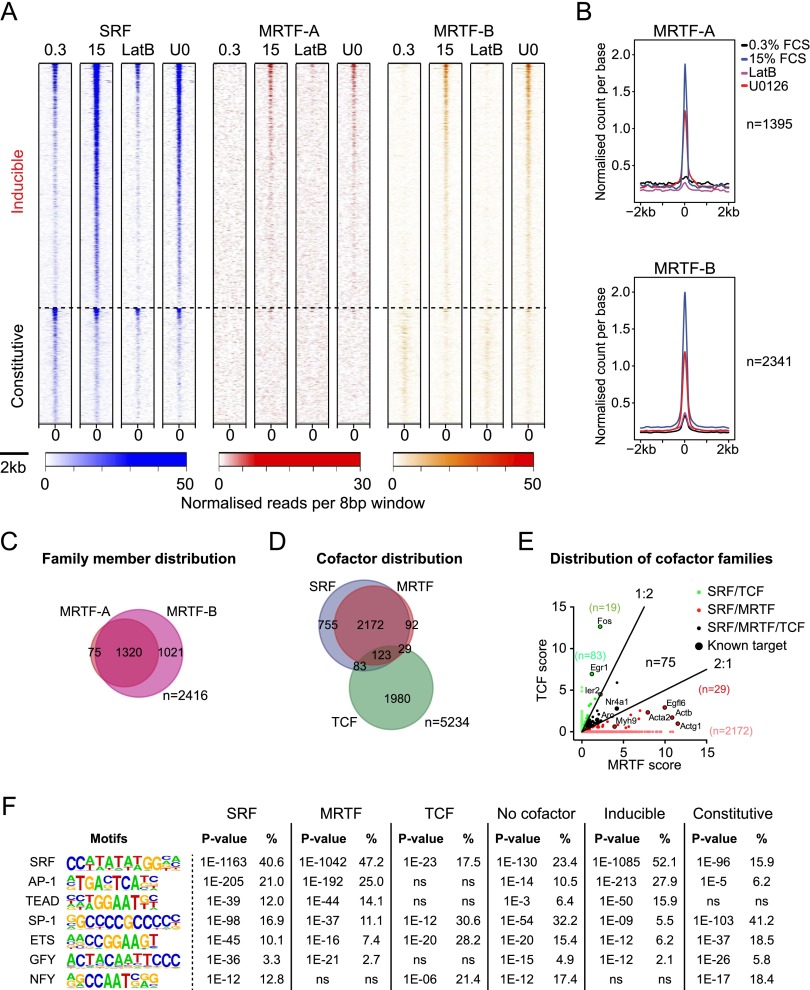
Gene-specific MRTF and TCF recruitment. (*A*) Heat maps showing correlation of SRF and MRTF binding at inducible and constitutive SRF sites across a 2-kb region centered on the SRF peak summit. Color intensity represents normalized reads per 8-bp window. See Supplemental Figure S2, D and E. (*B*) Metaprofiles of MRTF binding, centered on SRF peak summits. (*C*) Most MRTF-A sites detected in ChIP-seq bind MRTF-B. See Supplemental Figure S2F. (*D*) Venn diagram showing relationships between sites bound by SRF, either MRTF or any TCF. See Supplemental Figure S2, G and H. (*E*) Scatter plot showing relative binding of MRTFs and TCFs by binding score (see the Supplemental Material). Among the 123 genes binding both families, more than twofold difference in score is associated with preferential response to ERK or Rho signaling (Gineitis and Treisman 2001). (*F*) SRF-associated sequence motifs within 100 bp of SRF peak summits, classified by cofactor-binding and SRF-binding inducibility. For motifs associated with LatB-sensitive and LatB-insensitive “no-cofactor” SRF sites, see Supplemental Figure S2I.

We defined 2416 MRTF-binding sites using ChIP-seq (Figs. 1B, 2A; Supplemental Fig. S2C–G; Supplemental Table S1; see the Materials and Methods). MRTF binding was low but detectable in unstimulated cells and substantially increased upon serum stimulation, reflecting regulated MRTF nuclear accumulation; MRTF binding was sensitive to LatB but also exhibited a slight sensitivity to U0126 (Fig. 2A,B; Supplemental Fig. S2D). MRTF-A and MRTF-B form homodimers (Supplemental Fig. S2E). Both were detected at many sites (Fig. 2C), and the correlation between MRTF-A and MRTF-B raw read counts (Supplemental Fig. S2F) suggests that the apparently MRTF-B-specific peaks called in ChIP-seq reflect poor MRTF-A antibody quality rather than MRTF-B-specific binding. Indeed, MRTF-A binding was detectable at such sites by ChIP coupled with quantitative PCR (ChIP-qPCR) (Supplemental Fig. S2G). In contrast, MRTF read counts correlated only poorly at the 75 apparently MRTF-A-specific sites, and these warrant further investigation (Supplemental Fig. S2F). We also identified 121 MRTF sites where SRF was not detectable (Fig. 2D; Supplemental Table S1), of which 57 were associated with transcribed sequences and/or RNA polymerase II (Pol II) at inducible or constitutive genes, while 64 were well-defined orphan binding sites (Supplemental Fig. S3). Taken together, these data show that in NIH3T3 fibroblasts, the vast majority of MRTF sites are targeted by MRTF-A and MRTF-B homodimers or heterodimers, binding in association with SRF.

### MRTF and TCF recruitment is SRF site-specific

Inspection of the ChIP-seq data confirmed that at most binding sites, SRF exhibits a clear preference for either the TCF or MRTF family cofactors (Fig. 1B). We identified 2215 discrete TCF peaks, of which ∼10% colocalized with SRF (Fig. 2D,E; Supplemental Fig. S2H; Supplemental Table S1), a lower proportion than previously observed for the Elk-1 TCF (Boros et al. 2009). At the majority of SRF–MRTF sites, TCF binding was undetectable even under resting conditions, where MRTFs are cytoplasmic; conversely, MRTF binding was undetectable at many SRF–TCF sites even under induced conditions, when MRTFs are nuclear (Figs. 1B, 2D). Binding of both MRTFs and TCFs was detected at 123 SRF sites, but at 48 of these, one cofactor family or the other dominated (Fig. 2D,E). TCF-associated SRF binding was predominantly constitutive (Supplemental Table S1).

These results clearly show that SRF cofactor recruitment is SRF site-specific even though the TCF ChIP-seq data are of lower quality than the MRTF data. Using HOMER (Heinz et al. 2010), we investigated whether specific sequence motifs are associated with binding of the different cofactors. The AP-1 and TEAD motifs were preferentially associated with inducible, MRTF-associated sites, while SP-1, Ets, GFY, and NFY motifs were more strongly associated with constitutive, TCF-associated sites (Fig. 2F; see the Discussion). No cofactor binding was detected at 755 SRF-binding sites (Fig. 2D; Supplemental Table S1). Motif analysis of these “solo” sites revealed all of the motifs associated with the MRTF- and TCF-specific sites (Fig. 2F). Among the solo sites, 95 exhibited LatB-sensitive serum-inducible SRF binding and were enriched in motifs associated with MRTF–SRF sites, while the remainder were enriched in motifs associated with TCF–SRF sites (Supplemental Fig. S2I). Thus, although solo SRF sites may be targets of an as yet unidentified SRF cofactor, it remains possible that they represent MRTF and TCF targets not detected by ChIP-seq. More work is required to resolve this issue.

### SRF–MRTF cooperate to exclude nucleosomes at inducible binding sites

**Figure 3.**
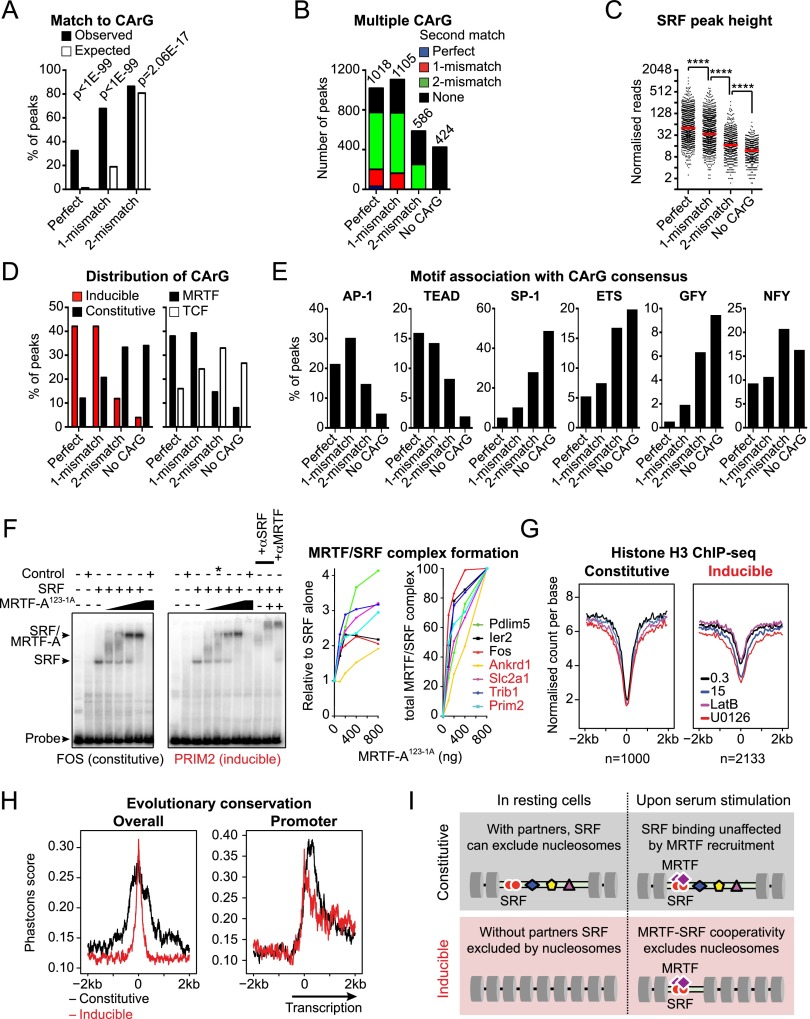
Characterization of MRTF- and TCF-associated SRF sites. (*A*) Match to the SRF CArG consensus (CCW_6_GG) within 100 bp of the SRF peak summit. Expected values are number of matches expected by chance with randomly selected sequences. (*B*) Multiple matches to the CArG consensus exist within 100 bp of each SRF peak summit. Peaks are grouped according to the best CArG match. (*C*) SRF peak height increases with matches to the CArG consensus. *P* < 0.0001, Mann-Whitney test. (*D*) Relationships between CArG consensus match and SRF-binding inducibility (*left*) or cofactor specificity (*right*). (*E*) SRF-associated sequence motif frequency plotted as function of match to the CArG consensus. (*F*) MRTF–SRF complex-binding properties at constitutive and inducible sites are similar in EMSA. (*Left*) EMSA analysis performed with whole-cell extract from NIH3T3 cells transfected with SRF expression plasmid (SRF) or vector alone (control) together with recombinant MRTF-A^123-1A^ (non-actin-binding mutant) (Vartiainen et al. 2007). An asterisk marks the binding conditions used for antibody supershift assays (αSRF and αMRTF). (*Right*) Yield of the total MRTF–SRF complex quantified relative to SRF alone, taken as 1 (*left* plot) or percentage of maximum (*right* panel). Inducible and constitutive SRF sites are coded red and black, respectively. (*G*) H3 ChIP-seq metaprofiles at constitutive and inducible SRF sites under different assay conditions. (*H*) Evolutionary conservation across SRF-binding sites, determined by the Phastcons algorithm. (*Left*) All sites. (*Right*) Promoter-associated sites (transcription at the *right*). (*I*) Indirect cooperativity model for inducible SRF binding. Constitutive SRF binding and low nucleosome occupancy are facilitated by low nucleosome affinity and/or binding of other transcription factors nearby, and MRTF activation therefore has no effect. Inducible SRF binding is associated with high nucleosome affinity and/or the absence of other transcription factor-binding events; at these sites, SRF binding alone is insufficient for nucleosome displacement, which requires formation of the MRTF/SRF complex.

To ascertain the basis for inducible SRF binding, we examined the binding site sequences in more detail. Almost 70% of SRF peaks contained perfect or singly mismatched copies of the SRF-binding CArG consensus sequence CC(AT)_6_GG (almost 90%, including double mismatches) (Fig. 3A), with many containing multiple copies (Fig. 3B); ChIP-seq peak heights correlated with the quality of the match to the CArG consensus (Fig. 3C). Surprisingly, inducible SRF-binding sites in general exhibited a better match to the CArG-box consensus than constitutive ones, and, similarly, MRTF-associated SRF peaks matched the CArG consensus better than TCF-linked ones (Fig. 3D). Consistent with this, the AP-1 and TEAD motifs were preferentially associated with strong CArG consensus matches, while the SP1, Ets, and GFY motifs were more prevalent at peaks with a poor or no match to the CArG consensus (Fig. 3E).

We examined MRTF–SRF complex formation in vitro by electrophoretic mobility shift assay (EMSA) using probe sequences derived from inducible and constitutive SRF sites (inducible: *Ankrd1*, *Slc2a1*, *Trib1*, and *Prim2*; constitutive: *Pdlim5*, *Ier2*, and *Fos*). Each probe bound SRF similarly in vitro even though SRF exhibited differential binding to these sites in vivo. Formation of the ternary MRTF-A–SRF complex resulted in a twofold to fourfold increase in DNA binding regardless of whether the complex was formed on a constitutive or inducible probe (Fig. 3F). Thus, signal-inducible binding of SRF in vivo cannot simply reflect cooperativity in the formation of the MRTF–SRF ternary complex.

We next investigated the relationship between SRF binding and nucleosome displacement using H3 ChIP-seq and ENCODE DNase I sensitivity data (GSM1003831) (The ENCODE Project Consortium 2011; Thurman et al. 2012). Both constitutive and inducible SRF-binding sites were associated with a local minimum in the H3 ChIP signal, which was more pronounced at constitutive SRF sites (Fig. 3G; Supplemental Fig. S4A). Consistent with this, SRF-binding sites colocalized with peaks of DNase I hypersensitivity (Thurman et al. 2012), with constitutive sites generally being more sensitive (Supplemental Fig. S4B,C). Serum stimulation significantly reduced H3 density at inducible but not constitutive SRF sites, and this was blocked by LatB, indicating it required MRTF activation (Fig. 3G; Supplemental Fig. S4A). These data suggest that inducible SRF binding reflects a requirement for MRTF–SRF complex formation in nucleosome displacement.

We reasoned that constitutive SRF binding might reflect cooperation between SRF and other transcription factors in nucleosome displacement (Thurman et al. 2012) and so evaluated evolutionary conservation around constitutive and inducible sites. The DNA sequences around constitutive SRF sites are evolutionarily conserved across a substantially wider region than those surrounding inducible SRF sites even when the latter are located in promoter regions (Fig. 3H), which are more evolutionarily conserved (The ENCODE Project Consortium 2007). Taken together, these data are consistent with a model in which SRF acts with partner transcription factors to displace nucleosomes at constitutive binding sites and with MRTF to displace nucleosomes at inducible binding sites (Fig. 3I; see the Discussion).

### SRF binding is associated with both active and serum-inducible genes

**Figure 4.**
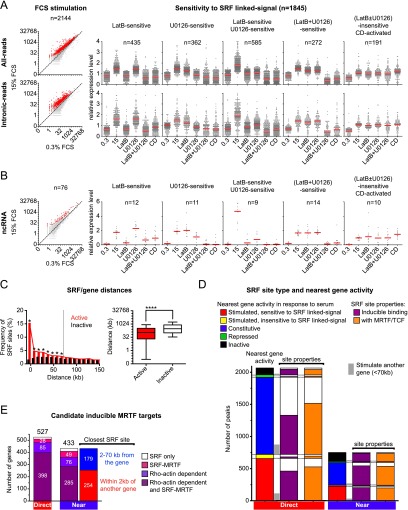
The majority of serum-inducible genes are MRTF-controlled. (*A*, *left*) Scatter plot display of total (*top*) and intronic (*bottom*) RNA-seq read counts before and after serum stimulation. Serum-stimulated genes (FDR = 0.2) are highlighted in red. (*Right*) Definition of serum-inducible genes sensitive to SRF-linked signal pathways (FDR = 0.08). (Red bars) Median. See Supplemental Table S2. (*B*) Signaling to ncRNA genes, as in *A*. See Supplemental Table S3. (*C*) SRF sites are overrepresented within 70 kb of transcriptionally active genes. (*Left*) Frequencies of SRF sites relative to active and inactive genes (per 10-kb bin relative to TSS or pA site). Zero indicates sites within 2 kb of the TSS or within a gene feature. (Asterisks) Significant at *P* < 0.05, multiple *t*-test with Holmes-Sidak correction. (*Right*) SRF-binding sites are significantly closer to active genes. (Asterisks) Significant at *P* < 0.0001, Mann-Whitney test. See Supplemental Table S4. (*D*) Relationship between SRF peak properties and activity of their closest associated gene. The *left* bars indicate peaks classified according to activity of the closest gene, gray bars at the side indicate the fraction of these peaks associated with regulatory events at genes up to 70-kb distant, the center bars represent classification of peaks by SRF-binding inducibility, and the right bars represent classification of peaks by cofactor association. (Direct) Sites within 2 kb of 5′ flanking sequences or within a gene feature; (near) sites within 70 kb of the TSS or pA site. See Supplemental Table S4. (*E*) Candidate MRTF serum-inducible genes (defined by MRTF binding, sensitivity to LatB or CD, or both) categorized by distance from the nearest SRF sites, as in *D*. Many inducible genes whose closest SRF site lies within 70 kb share that site with a second gene. See Supplemental Table S4A.

We used RNA sequencing (RNA-seq) to determine how transcription changes following a 30-min serum stimulation, analyzing both total RNA-seq reads and intronic RNA-seq reads to maximize our ability to detect gene expression changes. We first identified 2144 serum-inducible genes (Fig. 4A, left). We then defined genes potentially under SRF network control as those whose induction was significantly impaired by LatB and/or U0126 or that were also inducible by CD; this comparison identified 1845 of these as candidate SRF-linked inducible genes at a fold discovery rate (FDR) of 0.08 (Fig. 4A, right; Supplemental Table S2). A similar analysis identified 151 of 363 repressed genes identified as potential SRF-linked targets (Supplemental Table S2). In addition, we identified 56 noncoding transcripts sensitive to SRF-linked signals, including 13 pre-microRNAs (pre-miRNAs), 11 small nucleolar RNAs (snoRNAs), one small nuclear RNA (snRNA), and 31 long intergenic noncoding RNA (lincRNA) clusters (Fig. 4B; Supplemental Fig. S5C; Supplemental Table S3).

We next sought to define a maximum distance at which an SRF-binding site might be expected to influence transcription. SRF-binding sites were significantly more frequent within 70 kb of the 12,213 genes that were detectably transcribed in our cells (Fig. 4C; Supplemental Table S4A) and also enriched within active gene features, even beyond 70 kb from the TSS (*P* < 10^−4^, Fisher’s test). Among the 1845 candidate SRF-regulated genes, we therefore considered only those with SRF sites within 70 kb or within the gene feature as potential SRF targets, dividing them into two groups: “direct” genes with SRF sites within 2 kb 5′ to the TSS or within a gene feature and “near” genes within 70 kb of an SRF site.

Strikingly, of the 1976 direct genes, only 527 were induced by SRF-linked signals, while expression of 1242 apparently constitutive genes remained unchanged (Supplemental Table S4A). As a consequence, only 658 (∼32%) of the 2068 SRF-binding sites associated with direct genes were associated with inducible transcription. However, the signal dependence of SRF binding and SRF cofactor association was similar for both inducible and constitutive genes (Fig. 4D; Supplemental Table S4B; see the Discussion). Inducible genes in the near class were frequently associated with SRF sites that were closer to a second gene, which could be inducibly or constitutively transcribed (Fig. 4D,E; Supplemental Table S4B). Again, only ∼30% (226/752) of the SRF sites 2–70 kb distant from their nearest gene were associated with inducible transcription (Fig. 4D; Supplemental Table S4B). SRF binding was predominantly associated with serum-induced gene activation. Only 67 repressed genes within 70 kb of an SRF site appeared to be SRF-linked, compared with 960 that were activated (Supplemental Table S2).

### MRTFs regulate the majority of serum-inducible genes

The RNA-seq analysis above defines an SRF target gene set of 960 serum-inducible genes (527 direct and 433 near). More than 95% of these (921 genes) could be classified as candidate MRTF targets through MRTF ChIP, inhibition by LatB, activation by CD, or more than one of these criteria (Fig. 4E; Supplemental Fig. S5; Supplemental Table S4A), while a stringent MRTF–SRF-inducible gene set of 683 genes was defined as those that both bound MRTFs and were affected by the actin-binding drugs (Fig. 4E; Supplemental Table S5). Of the 33 SRF-linked ncRNA genes within 70 kb of an SRF site, all were candidate MRTF target genes (Supplemental Table S3). Finally, we defined a further set of 76 serum-inducible genes as high-confidence TCF–SRF targets based on TCF binding and sensitivity to U0126 (Supplemental Table S5).

### MRTF–SRF signaling promotes both Pol II recruitment and elongation

**Figure 5.**
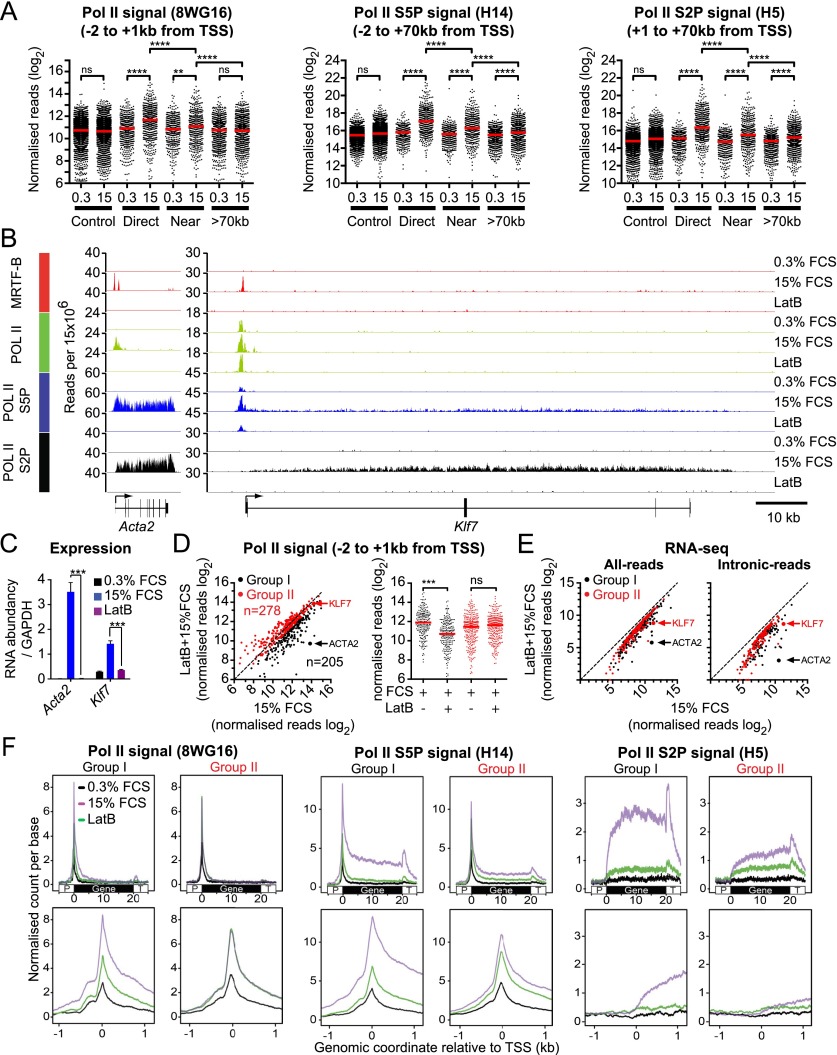
MRTF acts at Pol II recruitment and post-recruitment steps. (*A*) Serum-induced genes exhibit increased Pol II loading. RNA Pol II ChIP-seq analysis with antibodies as follows: 8WG16 (Pol II CTD un-P), total reads from −2 kb to +1 kb from TSS; H14 (Pol II CTD S5P), total reads from −2 kb to the gene 3′ end or +70 kb, the limit of Pol II progress after 30 min of stimulation; and H5 (Pol II CTD S2P), total reads from +1 to the gene 3′ end or +70 kb. Statistical significance, Mann-Whitney test, (**) *P* < 0.01; (****) *P* < 0.0001. (*B*) Representative MRTF-B and Pol II ChIP-seq tracks on *Acta2* and *Klf7*. (*C*) LatB inhibits serum induction of *Klf7* and *Acta2*, assessed by qRT–PCR. SEM of three independent experiments. (*D*) MRTF is required for Pol II recruitment on a subset of target genes. Four-hundred-eighty-three genes were analyzed whose serum-induced activation was LatB-sensitive in the absence of U0126. (*Left*) Scatter plot summary of the Pol II ChIP-seq signal from −2 to +1 kb around the TSS. Genes exhibiting >30% reduction (group I) are shown in black, and those exhibiting <30% reduction (group II) are in red. (*Right*) Summary of LatB’s effect on the two groups. Statistical significance, Mann-Whitney test, (***) *P* < 0.001. (*E*) LatB inhibits group I and group II gene RNA synthesis to a similar extent in RNA-seq. (*F*) Metaprofiles of Pol II ChIP-seq for group I and group II genes. (*Top*) 8WG16, H14, and H5 normalized ChIP-seq read counts are shown across gene loci, standardized to 20 kb, and flanking 5 kb. (*Bottom*) Read counts from −1 kb to +1 kb from the TSS.

Having defined the SRF target gene set by RNA-seq, we asked whether these genes exhibited changes in Pol II occupancy following serum stimulation using Pol II ChIP-seq. Serum-responsive genes exhibited significantly increased Pol II loading following serum stimulation, the increase being maximal for the direct genes (Fig. 5A; see the Discussion). We exploited this observation to investigate at which step MRTFs act in the transcription cycle by examining the effect of LatB on Pol II recruitment at 483 SRF target genes whose serum induction was sensitive to LatB.

Examination of individual ChIP-seq profiles revealed that at genes such as *Acta2*, Pol II recruitment to the vicinity of the TSS was substantially serum-inducible and LatB-sensitive, while genes such as *Klf7* exhibited substantial Pol II recruitment prior to MRTF activation (Fig. 5B). In both cases, however, production of mature RNA was sensitive to LatB and therefore was MRTF-dependent (Fig. 5C). We examined the effect of LatB on Pol II recruitment at the promoter region (−2 kb/+1 kb relative to the TSS) across the whole population. Setting a threshold of >30% for the LatB-induced decrease in Pol II ChIP-seq reads allowed definition of two categories of MRTF target gene: Pol II recruitment to the 205 group I genes was LatB-sensitive, while Pol II recruitment to the 278 group II genes was LatB-insensitive (Fig. 5D). Nevertheless, both groups exhibited similar sensitivity to LatB in RNA-seq analysis (Fig. 5E). Group I genes exhibited a greater degree of inducibility, as assessed by both intronic RNA-seq reads and Pol II density. In both groups, Ser5 and Ser2 phosphorylated Pol II C-terminal domain (CTD) ChIP-seq signals within the gene body were LatB-sensitive (Fig. 5F). Taken together, these data indicate that MRTF activation facilitates both Pol II recruitment and promoter escape according to gene context (see the Discussion).

### Ontology of MRTF–SRF and TCF–SRF target gene sets

**Figure 6.**
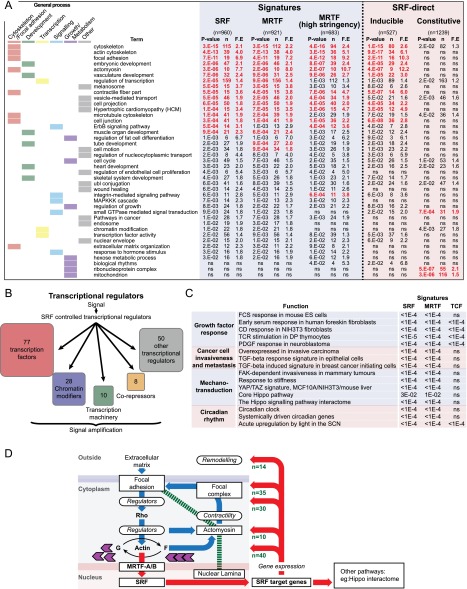
GO analysis of SRF targets. (*A*) SRF- and MRTF-linked gene signatures were analyzed using DAVID. (SRF) Serum-inducible genes with an SRF-binding site within 70 kb; (MRTF) serum-inducible genes with MRTF–SRF binding within 70 kb or LatB and/or CD sensitivity; (stringent MRTF) MRTF–SRF binding within 70 kb and sensitive to LatB or CD. The signatures are compared with SRF-linked serum-inducible or constitutively transcribed “direct” target genes (i.e., with SRF sites within 2 kb of a 5′ flanking sequence or within a gene feature). See also Supplemental Tables S6 and S7. (F.E.) Fold enrichment. (*B*) SRF-controlled genes involved in transcriptional regulation are subdivided into functional categories. (*C*) Relationship between the inducible SRF, MRTF, and TCF gene signatures and previously defined sets of genes up-regulated between two experimental conditions; statistical significance by two-tailed Fisher test. See Supplemental Table S8. (*D*) MRTF–SRF signaling is a nuclear component of integrin-mediated “inside-out” signaling. Classes of SRF target genes involved in adhesion signaling and mechanosensation are shown. Engagement with the ECM induces changes in actin dynamics and actomyosin contractility, promoting focal adhesion assembly (blue arrows). MRTF–SRF target gene expression provides an additional long-term “inside-out” signaling mechanism (red arrows). The green dashed line indicates direct physical coupling between actomyosin, focal adhesions, and the nucleus.

Gene ontology (GO) analysis of the MRTF–SRF target genes using DAVID (Huang et al. 2009) revealed hundreds of genes involved in actin filament dynamics, cell adhesion, extracellular matrix (ECM) synthesis and processing, cell motility, and other actin-linked processes as well as a significant number of genes involved in microtubule-based cytoskeletal dynamics (Fig. 6A; Supplemental Fig. S6A; Supplemental Tables S6, S7). Consistent with this, SRF inactivation was associated with defects in the F-actin and microtubule cytoskeletons and increased nuclear size and aspect ratio (Supplemental Fig. S6B). A second major MRTF–SRF target class includes transcriptional machinery, chromatin regulators, and >70 transcription factors, including the classical AP1 and Egr families and regulators of differentiation, cell morphogenesis, and motility (Fig. 6A,B). The SRF target gene set is also enriched in genes involved in cell growth and metabolism, including circadian clock components, and genes controlled by systemic circadian cues (Fig. 6A,C; for references, see Supplemental Table S8).

MRTF–SRF target genes overlap with gene signatures associated with cancer cell invasiveness and metastasis, response to ECM stiffness, or response to FAK or TGFβ signaling (Fig. 6C,D; for references, see Supplemental Table S8). In several cases, the inducible MRTF–SRF target gene set exhibited significant overlap with gene sets identified as both up-regulated and down-regulated between two experimental conditions. We feel this likely reflects the normalization procedures used for comparative analysis of microarray data sets, which generally assume similar overall RNA expression levels between conditions (for discussion, see Loven et al. 2012). The MRTF and YAP–TAZ signatures also overlap significantly (Fig. 6C; Supplemental Table S8) even though YAP, which is also a target for Rho signaling (Yu et al. 2012) appears to be constitutively active in our experimental conditions (Supplemental Fig. S1C–E). The MRTF–SRF signature also includes many components of the Hippo signaling interactome (Fig. 6C; for references, see Supplemental Table S8). The TCF-dependent signature showed significant overlap with HeLa cell Elk1–SRF targets previously defined by ChIP–chip, TCF-dependent TCR-activated genes in thymocytes, and ERK-dependent genes activated by PDGF in fibroblasts or light in the suprachiasmatic nucleus (SCN) (Fig. 6C; Supplemental Table S8).

Constitutively active direct genes exhibited enrichment for genes involved in metabolism, DNA synthesis, gene expression, and cell growth (Fig. 6A; Supplemental Table S6) and were functionally different from inducible genes, according to fold enrichment of individual GO terms (Wilcoxon test *P* < 0.0001, for “direct” gene subsets). The failure of these genes to respond to signals despite their association with SRF cofactors suggests that they are somehow refractory to SRF-linked signals (see the Discussion).

### Both MRTF- and TCF-linked signaling to SRF can resynchronize the circadian clock

**Figure 7.**
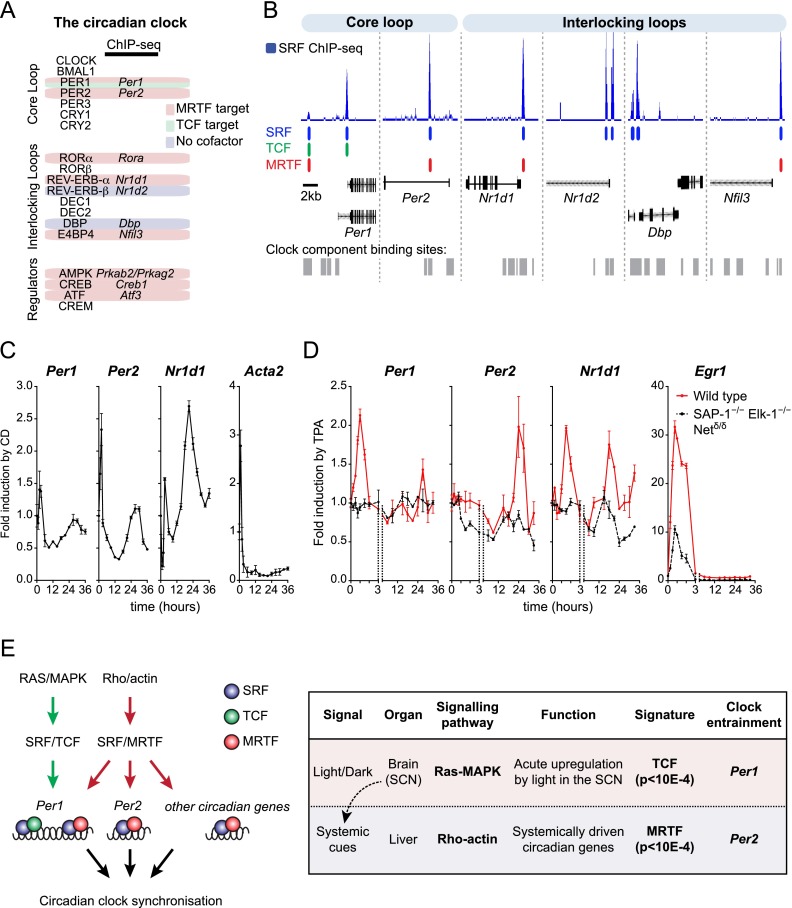
SRF and MRTF can both reset the circadian clock. (*A*) SRF targets among circadian clock circuits (for discussion of regulatory loops, see Koike et al. 2012). (*B*) SRF, MRTF, and TCF ChIP-seq peaks are shown *below* in blue, red, and green, respectively, aligned with binding sites in the liver for the clock genes *Per1* and *Per2* (core loop components) and *Nr1d1, Nr1d2, Dbp*, and *Nfil3* (interlocking loop components) (Koike et al. 2012), shown in gray. (*C*) Clock resetting by MRTF activation. NIH3T3 cells were treated with 2 µM CD for 2 h followed by washout; transcripts were quantified by qRT–PCR over 36 h. For serum stimulation kinetics, see Supplemental Figure S7C. (*D*) Clock resetting by TCF activation. Wild-type and SAP-1^−/−^ Elk1^−/−^ Net^∂/∂^ MEFs were treated with TPA, and transcripts were quantified by qRT–PCR. (*E*) Circadian clock synchronization by MRTF- and TCF-linked SRF signaling.

Identification of components of the circadian clock among SRF target genes was intriguing given that serum shock can reset the circadian clock in fibroblasts (Balsalobre et al. 1998). Of SRF targets in the core clock network, *Per1*, *Per2*, *Nr1d1*, *Rora*, and *Nfil3* bound MRTFs, but only *Per1* bound the TCFs (Fig. 7A,B; Supplemental Fig. S7A; for references, see Supplemental Table S8). Similar to serum shock, MRTF activation by CD was sufficient to resynchronize the fibroblast clock (Fig. 7C; Supplemental Fig. S7B,C), and this required SRF (Supplemental Fig. S7D). We have shown elsewhere that systemic circadian activation of SRF target genes in the liver is associated with circadian fluctuations in MRTF activity (Gerber et al. 2013).

MAPK signaling induced by TPA was previously shown also to be sufficient to reset the fibroblast clock (Akashi and Nishida 2000). Since *Per1* is the only core clock component that recruits TCFs, we tested whether it was sufficient for clock resetting. In mouse embryonic fibroblasts (MEFs), TPA stimulation rapidly activated *Per1* but not *Per2*; however, *Per2* and other core clock components were also activated 24 h later (Fig. 7D). Both the immediate and longer-term transcriptional activation of clock transcription were abolished in MEFs lacking all three TCFs (Fig. 7D). Taken together, these data show that MAPK-induced resetting of the circadian clock is mediated by TCF–SRF signaling to *Per1* in fibroblasts. Thus, *Per1* and *Per2* represent SRF target genes linking the core clock components to two different SRF-linked signal pathways, Ras–ERK–TCF and Rho-actin–MRTF, which are triggered by different systemic and cell-specific cues (Fig. 7E).

## Discussion

In this study, we showed that Rho-actin signaling to the MRTF family of SRF coactivators regulates multiple aspects of the transcriptional response to serum stimulation in fibroblasts. We identified ∼3100 binding sites for SRF, associated with ∼2600 genes. SRF acts as principal gene targeting factor for the MRTFs, and much of SRF binding is controlled through MRTF recruitment, which facilitates nucleosome displacement. The majority of 960 serum-inducible candidate SRF target genes that we identified are regulated through MRTF–SRF signaling. MRTF–SRF target genes include hundreds involved in actin cytoskeletal structures and dynamics, suggesting that MRTF–SRF signaling constitutes a long-term aspect of the “inside-out” cellular response to adhesion. Finally, at the transcriptional level, MRTF–SRF signaling potentiates both RNA Pol II recruitment and promoter escape according to gene context.

### SRF cofactor recruitment is gene-specific

We found that a substantial majority of the SRF sites in fibroblasts recruit both MRTFs, with only a minority of sites binding TCFs. In general, SRF sites exhibited a clear preference for one cofactor family or the other, with only a small number of sites binding both at comparable levels. Thus, SRF cofactor recruitment is gene-specific, consistent with predictions from functional studies of model genes (Gineitis and Treisman 2001) and biochemical experiments (Miralles et al. 2003; Wang et al. 2004; Zaromytidou et al. 2006). MRTF-A and MRTF-B form heterodimers, and both were detectable at the majority of MRTF sites. SRF appears to be the primary targeting agent for the MRTFs in fibroblasts: Ninety-five percent of 2416 MRTF-binding events detected in fibroblasts were SRF-associated, and we found no evidence for MRTF recruitment by other sequence-specific DNA-binding proteins as proposed by others (Qiu et al. 2005; Morita et al. 2007). The functional significance of the small number of apparently SRF-independent MRTF–DNA interactions and a small number of apparently MRTF-A-specific binding events remains unclear; these include associations with transcribed sequences themselves and a small number of intergenic sequences. Cofactor binding could not be detected at ∼25% of SRF sites. At these sites, SRF might act with hitherto undetected cofactors or perhaps constitutively activate transcription through its C-terminal transcription activation domain (Johansen and Prywes 1993); however, examination of the sequence motifs associated with them suggests that they might also represent undetected MRTF- or TCF-associated sites (see below).

Our data demonstrate cofactor-specific association between SRF binding and other transcription factor motifs. The AP-1 or TEAD motifs are frequently found at MRTF-specific SRF sites, and it is conceivable that the MRTFs interact directly with these: The MRTFs are known to make DNA contacts, and their SAP domains have been implicated in promoter selectivity (Wang et al. 2001; Zaromytidou et al. 2006). Alternatively, the TEAD and AP-1 motifs may reflect functional cooperation between MRTFs and their cognate transcription factors, and, in this respect, the TEAD motifs are particularly intriguing given the apparent overlap between YAP–TAZ and MRTF–SRF target genes (see below). In contrast, we found that the Ets, NFY, and SP1 motifs, previously demonstrated to be SRF-associated (Valouev et al. 2008; Sullivan et al. 2011), were associated specifically with TCF–SRF-binding events. As discussed below, the enrichment of these motifs at constitutive SRF sites with poor matches to the CArG-binding consensus may reflect cooperativity between SRF and their cognate factors in nucleosome displacement.

### Most MRTF–SRF binding is signal-regulated

A substantial majority of SRF-binding events are potentiated by MRTF activation, a surprising finding given classical genomic footprinting studies (Herrera et al. 1989), although inducible binding has been reported more recently (Kim et al. 2010; Leitner et al. 2011). Our in vitro MRTF–SRF complex formation experiments and observation that inducible SRF sites are better matched to the binding consensus than constitutive ones show that inducible SRF binding cannot simply reflect recruitment of SRF to weak binding sites by cooperativity with MRTF. Instead, we found that MRTF–SRF complex formation induces nucleosome displacement at inducible SRF-binding sites but that constitutive SRF sites remain unaffected.

We propose that constitutive SRF binding reflects cooperation with transcription factors binding nearby to induce nucleosome displacement even under resting conditions. Previous studies have shown that nucleosome displacement is facilitated by multiple independent transcription factor-binding events (Boyes and Felsenfeld 1996), while at transcription factor clusters, aggregate ChIP-seq peak heights correlate with DNase I sensitivity (Thurman et al. 2012). Consistent with this, constitutive SRF sites are embedded in broad regions of evolutionary conservation with a greater frequency of associated binding motifs. In contrast, inducible sites are found in narrower regions of conservation, suggesting that cooperating transcription factors are less abundant and that it is MRTF recruitment that increases the probability of nucleosome displacement (Fig. 3I).

### SRF is associated with transcriptionally active genes

SRF-binding sites are preferentially associated with transcriptionally active genes, the majority being located within gene features. We exploited this and the known involvement of SRF with serum-regulated transcription to define an SRF target gene set comprising the 960 serum-inducible genes within 70 kb of an SRF site, almost 90% of which were dependent on SRF cofactor-linked signal pathways for transcription. Among these, we identified an MRTF–SRF target gene set of 921 genes, as assessed by MRTF ChIP and/or sensitivity to MRTF-linked signals, and a stringent set of 683 MRTF–SRF targets satisfying both criteria. We also identified a 76-gene TCF–SRF-inducible gene signature based on TCF binding and U0126 sensitivity. Analysis of Pol II loading on serum-inducible genes showed a steady decrease of distance from the nearest SRF site, suggesting that at the time point that we analyzed, the primary determinant of transcription rate is SRF activity.

Our data show that SRF is overwhelmingly associated with serum-induced transcriptional activation. Although >14 times as many genes were activated as were repressed, the molecular basis of repression should be studied further. More significantly, we found that more than two-thirds of the genes associated with SRF sites are apparently constitutively transcribed. These genes, which are ontologically distinct from the serum-inducible SRF target set, also bind SRF cofactors but show no response to serum stimulation even though the SRF network is maximally active at the 30-min time point analyzed. Indeed, examination of SRF targets expressed in both NIH3T3 cells and MEFs revealed that only 8% of the constitutively active SRF-linked genes were dependent on SRF, compared with 56% of the shared serum-inducible genes (C Esnault and R Treisman, unpubl.). Promoter-proximal SRF binding was previously observed in macrophages and, similarly, expression of only 6% of these genes was dependent on SRF (Sullivan et al. 2011). It is likely that constitutive SRF-linked promoters are somehow refractory to transcriptional activation, although we cannot exclude the possibility that they become active at late times, and it will be interesting to determine the molecular basis for this.

### MRTF and transcription activation

Members of the TCF family of SRF cofactors interact with MED23 and other mediator subunits to promote Pol II recruitment and promoter escape at TCF–SRF target genes such as *Egr1* (for references, see Balamotis et al. 2009). Our RNA Pol II ChIP-seq data indicate that while active MRTF is required for both RNA Pol II recruitment and activation at about half of its targets, it is required for only a post-recruitment step at the others. Thus, in addition to promoting Pol II recruitment, the MRTFs also act at a post-recruitment step in transcriptional activation. We previously reported that confinement of MRTFs to the nucleus in resting cells does not activate transcription (Vartiainen et al. 2007) and are currently investigating the relationship between actin–MRTF interaction and the different steps of transcription activation.

### Functional significance of MRTF–SRF and TCF–SRF target gene sets

Our results reveal that in fibroblasts, it is MRTF–SRF signaling that underlies the role of SRF in cytoskeletal dynamics. Within the 921-gene MRTF–SRF signature, gene targets include components of both the actin and microtubule cytoskeletons, cell–cell and cell matrix junctions, ECM components, and vesicle trafficking components. The involvement of Rho-actin signaling in microtubule-associated gene expression is perhaps not surprising given the role of Rho GTPase signaling in the response to challenge to microtubule integrity (for discussion, see Krendel et al. 2002). The MRTF-dependent serum response also includes numerous transcriptional regulatory factors and a significant number of genes involved in cell growth and metabolism, including many of the components of the core circadian clock network.

### SRF in circadian clock regulation

Previous work has shown that in fibroblasts, both serum stimulation and ERK activation can reset the circadian clock (Balsalobre et al. 1998; Akashi and Nishida 2000). Our results show that the core clock circuit genes are SRF targets and that both MRTF- and TCF-linked signals can reset the clock. We have shown elsewhere that systemic circadian cues promote oscillations of G-actin concentration that control MRTF–SRF signaling in the liver (Gerber et al. 2013). Here we showed that in fibroblasts, only *Per1* is a specific TCF–SRF target gene, and ERK activation induces an immediate transcriptional response of *Per1*, resynchronizing the core clock in a TCF-dependent manner. MAPK signaling has been previously shown to induce immediate–early gene expression and reset the clock in the SCN (Obrietan et al. 1998). Our data suggest that SCN clock resetting will reflect TCF-dependent *Per1* activation: Indeed, the TCF-dependent gene signature strongly overlaps with that of light-induced gene transcription in the SCN, which induces *Per1* but not *Per2* transcription.

### MRTF–SRF signaling and mechanosensing

The MRTF–SRF gene signature significantly overlaps with gene signatures characteristic of cancer cell invasion and metastasis and the response to mechanical stress. Integrin engagement with the ECM leads to FA assembly, requiring Rho-dependent actomyosin contractility, and this process, known as inside-out signaling, is strongly implicated in cancer cell invasiveness (Paszek et al. 2005; for overview, see Butcher et al. 2009). Our data show that MRTF–SRF pathway activation constitutes an additional long-term nuclear arm of inside-out signaling, controlling expression of the major FA force sensors p130Cas/*Bcar1*, Talin/*Tln1*, and Vinculin/*Vcl* and many actomyosin components and regulators (Fig. 6D; Supplemental Table S7). An additional aspect of MRTF–SRF inside-out signaling is its potential effects on the ECM itself, since it controls expression of collagens, their modifiers (*Lox* and *Plod3*), and matrix metalloproteases (*mmp9* and *serpine1*) (Fig. 6D; Supplemental Table S7). Indeed, *Lox* expression promotes FAK-dependent matrix stiffening, FA assembly, and invasiveness (for review, see Barker et al. 2012).

Matrix stiffness is an important determinant of mechanical stress that induces MRTF activation (Huang et al. 2012), and the MRTF–SRF signature overlaps significantly with the matrix stiffness-associated breast cancer invasiveness and FAK-dependent gene signatures (Provenzano et al. 2008, 2009). Matrix stiffness enhances osteogenic differentiation while inhibiting adipogenesis (for review, see Butcher et al. 2009; Discher et al. 2009); consistent with this, Rho signaling to SRF promotes osteogenesis and inhibits adipogenic differentiation (Sordella et al. 2003; Mikkelsen et al. 2010; Chen et al. 2012). Matrix stiffening also induces increased expression of *Lmna*, an SRF target, which is required for effective differentiation along muscle and bone lineages (Swift et al. 2013) and stiffening of the nucleus itself (Lammerding et al. 2006). Interestingly, *Lmna* inactivation results in increased G-actin levels and decreased MRTF activity, probably through deregulation of emerin (Ho et al. 2013). The relationship between matrix stiffness, G-actin concentration, and MRTF activity deserves systematic investigation.

### SRF signaling and the YAP–TAZ pathway

The YAP–TAZ pathway is also mechanoresponsive but is thought to be independent of MRTF–SRF signaling (Dupont et al. 2011; Calvo et al. 2013). Although in our experiments YAP is nuclear and presumably active, our MRTF–SRF signature overlaps significantly with those published for YAP and includes *Ctgf*, *Cyr61*, and *Ankrd1*, genes frequently used as readouts for YAP activation. Moreover, MRTF–SRF signaling may also influence YAP activity indirectly, as the MRTF signature includes YAP–TAZ pathway and Hippo interactome components (Kwon et al. 2013), including TAZ/*Wwtr1*, *Tead1*, and *Runx2* (Fig. 6C; Supplemental Table S8). These observations and the occurrence of the TEAD motif at MRTF–SRF sites suggest that the relationship between YAP/TAZ and MRTF signaling should be investigated further.

### SRF signaling and ncRNAs

We identified 33 serum-inducible ncRNAs as SRF targets, including miRNAs, snoRNAs, and long ncRNAs (lncRNAs). Where known, the properties of these ncRNAs suggest that they may also contribute to the serum response (Supplemental Fig. S5C). For example, miR-143 and miR-145 affect cytoskeletal dynamics in SMCs, miR-199a2 and miR-214 control myoblast differentiation, and miR-21 promotes fibrosis and epithelial-to-mesenchymal transition (EMT) and is implicated in cancer, while miR-22 is implicated in regulation of SRF itself in a cardiac overload model. The lncRNAs controlled by MRTF–SRF signaling include Tug1 and Malat1/Neat2 (a lncRNA implicated in lung cancer cell invasion and metastasis), which are recruited by unmethylated and methylated PC2, respectively. Intriguingly, PC2 itself and KDM4C, the PC2 demethylase, are also MRTF targets. Further work will be required to establish their function during serum stimulation. Finally, it will be interesting to investigate whether SRF-dependent lncRNAs such as GM15270/*Ctgf* and GM13270/*Rsu1* are involved in the regulation of SRF-controlled protein-coding genes in their vicinity.

### Concluding remarks

MRTF–SRF signaling is an important aspect of the response to growth factor signaling and an important mediator of the cytoskeletal response to Rho GTPase activation. The MRTF–SRF will provide an interesting test bed for investigation of the relationships between cell–cell and cell–substrate interactions, cytoskeletal dynamics, and gene expression.

## Materials and Methods

### ChIP and ChIP-seq

ChIP was performed as described (Miralles et al. 2003; Costello et al. 2010) with minor modifications. Additional antibodies used were MRTF-A (sc-21558), MRTF-B (sc-47282), Pol II CTD S2unP, and 8WG16 (sc-56767), all from Santa Cruz Biotechnology; total H3 (ab1791, Abcam); Pol II CTD S2P (H5, Covance); and Pol II CTD S5P (H14, Covance). See the Supplemental Material.

### ChIP-seq peak calling strategy

#### SRF sites

Two independent chromatin preparations from resting and serum-stimulated cells and single preparations from cells stimulated in the presence of inhibitors were used for ChIP-seq with a pooled bead-alone control. Peaks were called using a stringent MACS threshold of *P* < 10^−5^ (Zhang et al. 2008). A core set of 3040 sites was defined as sites detected in both resting samples + sites detected in both stimulated samples + sites detected in a LatB sample and any other sample + sites detected in a U0126 sample and any other sample. An additional 93 SRF-binding sites were defined on the basis that they coincided with MRTF peaks called by MACS at *P* < 10^−5^, were serum-inducible and LatB-sensitive, and passed a low MACS threshold of *P* < 0.05. Constitutive and inducible SRF sites were defined using an inducibility threshold (SRF signal in 15% FCS/signal in 0.3% FCS) such that the linear regression curve was closest to 1 for the constitutive SRF population, which by definition should not be influenced by signal.

#### MRTF-A and MRTF-B

Single ChIPs from resting cells and serum-stimulated cells with or without LatB or U0126 were analyzed. Two-hundred-seventy-four MRTF-A and 1178 MRTF-B peaks were called by MACS at *P* < 10^−5^ and detected (1) in more than one sample for each antibody or (2) in the same condition by both MRTF-A and MRTF-B antibodies, since MRTF-A and MRTF-B heterodimerize (Supplemental Fig. S2E). A further 1121 MRTF-A and 1165 MRTF-B peaks were called as coincident with an inducible, LatB-sensitive SRF site at MACS *P* < 0.05, since SRF and MRTF read counts correlate well at inducible SRF peaks (Supplemental Fig. S2C). MRTF-A and MRTF-B read counts strongly correlate at sites called for both proteins (Supplemental Fig. S2F, left) and those called specifically for MRTF-B (Supplemental Fig. S2F, center), suggesting that failure to detect MRTF-A- and MRTF-B-specific sites generally reflects MRTF-A antibody quality; indeed, MRTF-A binding could be detected at such sites by conventional ChIP-qPCR (Supplemental Fig. S2G).

#### TCFs—SAP-1, Elk-1, and Net

We analyzed a single ChIP from resting cells or serum-stimulated cells with or without LatB or U0126. Peaks were called at MACS *P* < 10^−5^ as those exhibiting signal in (1) more than one condition for the same family member or (2) for multiple family members in the same condition.

#### RNA Pol II

Pol II signal was quantified for all RefSeq genes and their promoters. Enrichment was determined counting the number of normalized reads in 500-base-pair (bp) windows from −2 kb to +70 kb from the TSS or to the gene end if shorter. Metaprofiles were generated as nucleotide average read density for the Pol II data sets across gene loci, standardized to 20 kb (±5 kb).

#### H3

Read counts were normalized to a total of 300 million reads, and the normalized read density per base pair was calculated. Metaprofiles were average read density profiles centered on the SRF peak summits.

### RNA

Total RNA was isolated using GenElute kit (Sigma). For qPCR quantitation, 500 ng of RNA was subjected to cDNA synthesis using the SuperScript III first strand synthesis system and random hexamer primers (Invitrogen). Data are from at least three independent experiments. Libraries were prepared using the Directional mRNA-seq Library Preparation version 1.0 prerelease protocol (Illumina) with minor adjustments. To minimize the ribosomal content, we used DSN (Evrogen JSC) or Ribozero (Epicentre) treatment.

### RNA-seq

After RNA library preparation and sequencing, we screened the raw data against protein-coding gene and ncRNA gene databases (RefSeq [release 47] and Ensembl [release 69], respectively) and derived data sets comprising either total read count across a given gene (“all reads”) or within introns only (“intronic reads”). Expression levels in unstimulated cells were fitted to a Gaussian, and 6664 genes were selected whose expression following stimulation differed by no more than 1 SD from the mean in unstimulated cells. The mean expression level of these 6664 genes was then used to normalize read counts between different experimental conditions. Differential expression analysis was performed with DESeq (Anders and Huber 2010) at an estimated FDR of <0.08. The serum-stimulated gene set was defined as those genes whose normalized “all reads” and/or “intronic reads” expression changes significantly. For details, see the Supplemental Material.

### Data access

ChIP-seq and RNA-seq data are available under Gene Expression Omnibus accession number GSE45888.
